# A Two-Decade-Delayed Diagnosis of Hereditary Angioedema: A Case Report

**DOI:** 10.7759/cureus.85019

**Published:** 2025-05-29

**Authors:** Swarup Shrestha, Ajay K Yadav, Dibas Khadka, Ramila Shrestha, Mukesh S Paudel

**Affiliations:** 1 Department of Gastroenterology, Bir Hospital, National Academy for Medical Sciences (NAMS), Kathmandu, NPL

**Keywords:** family history, genetic disorder, hereditary angioedema, recurrent abdominal pain, relapsing and remitting

## Abstract

Hereditary angioedema (HAE) is a rare genetic disorder characterized by recurrent episodes of angioedema that can affect the skin, gastrointestinal tract, and upper respiratory tract. Angioedema of the gastrointestinal tract can manifest as recurrent episodes of abdominal pain, which can often be misdiagnosed as a functional bowel disorder, a neuropsychiatric condition, or even an acute abdomen, which may result in unnecessary investigations and treatment, including surgeries. Angioedema of the skin can present as non-pitting swelling in the limbs and face. In contrast, when the respiratory tract is involved, it can sometimes lead to laryngeal edema, which can be life-threatening if not treated promptly. Diagnosing HAE can be particularly difficult for individuals without a family history of the condition. We present the case of a 30-year-old female patient who was diagnosed with HAE two decades after her initial symptoms appeared, which caused her significant suffering during that time without any targeted therapy. After her diagnosis, she received treatment with a plasma-derived C1-esterase inhibitor (C1-INH), which successfully decreased the frequency and severity of her symptoms and thus alleviated her suffering.

## Introduction

Hereditary angioedema (HAE) is a rare genetic disorder caused by a dysfunction or complete absence of the C1 esterase inhibitor (C1-INH) gene. Although HAE is classified as a rare autosomal dominant disease, about 25% of HAE cases are sporadic, caused by de novo mutations. HAE is a multisystemic disease affecting various organs, including the skin, gastrointestinal tract, and upper respiratory tract [1]. This case discusses a 30-year-old Nepali woman of Aryan descent who presented with recurrent abdominal pain. Some episodes were also associated with swelling of the extremities and face, while a few episodes included laryngeal edema. After two decades of suffering, she was finally diagnosed with hereditary angioedema. This case is notable due to the absence of family history and a long duration of symptoms before diagnosis, highlighting the importance of early diagnosis and effective management of such rare cases.

## Case presentation

**Figure 1:**
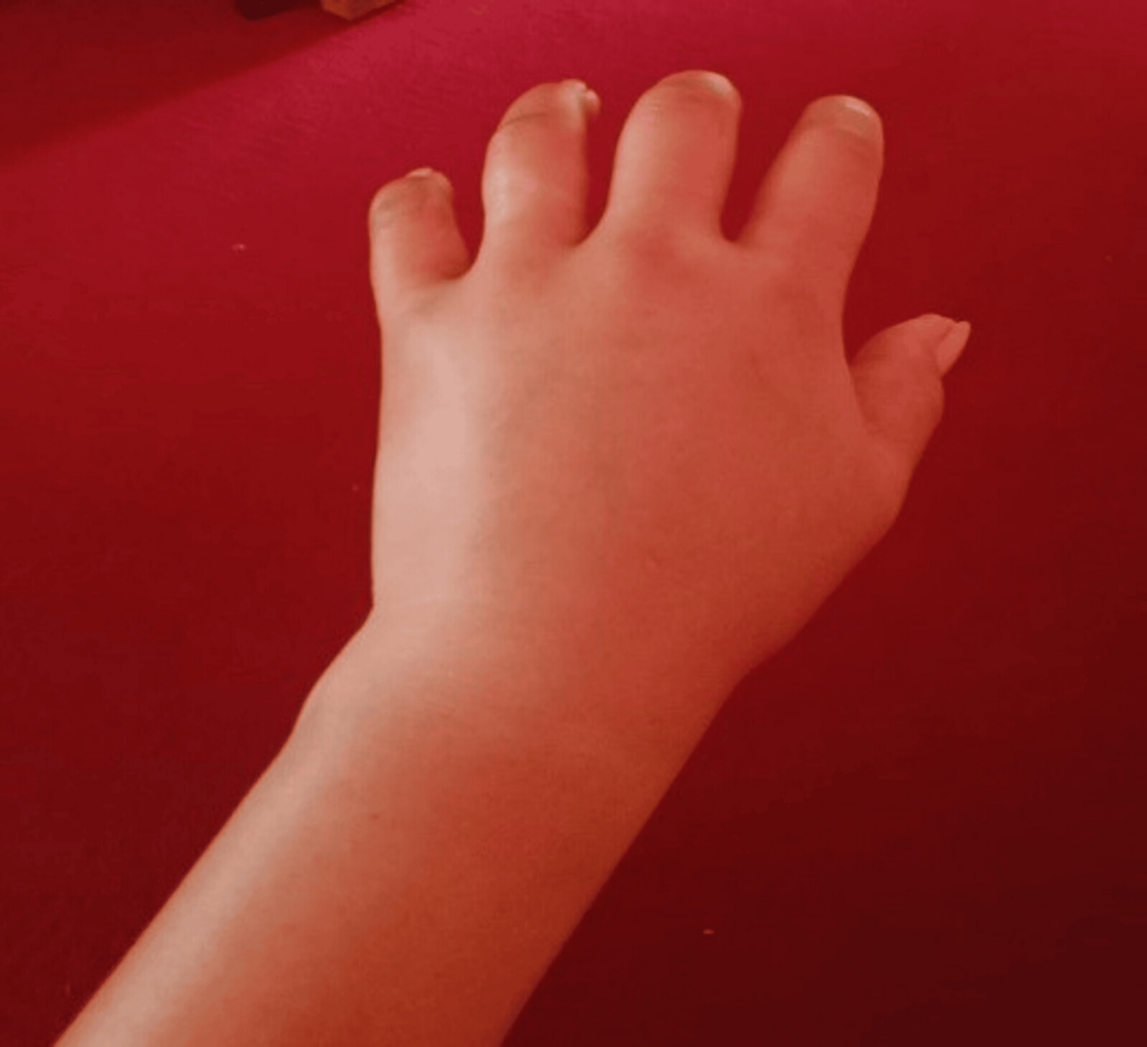
Non-pitting edema of the left upper limb Photo taken eight weeks prior, during the acute episode.

**Figure 2:**
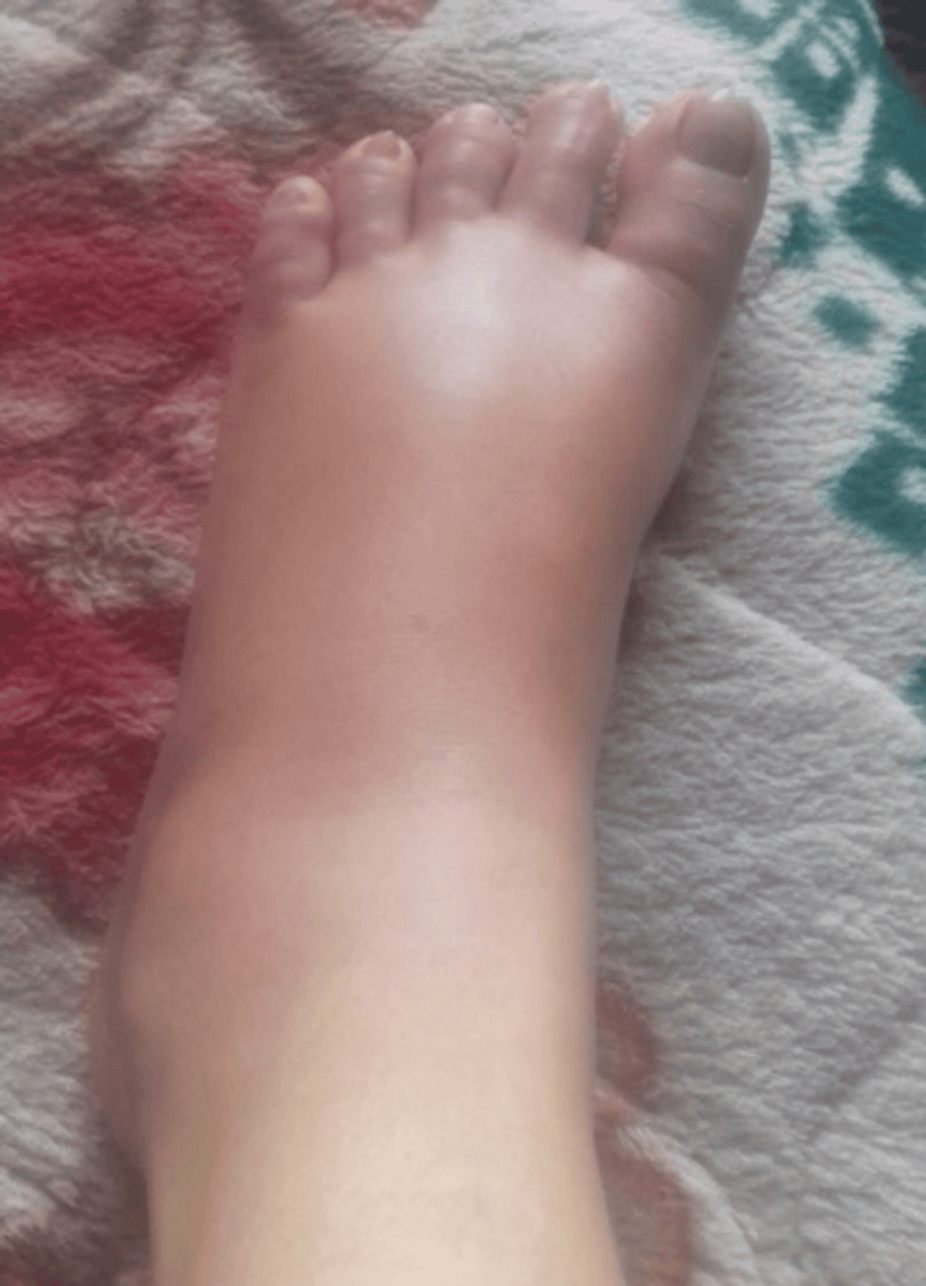
Non-pitting edema of the left lower limb Photo taken eight weeks prior, during the acute episode.

**Figure 3:**
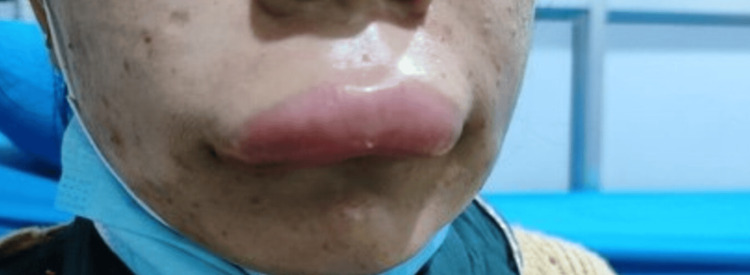
Swelling of the face and upper lip Photo taken eight weeks prior, during the acute episode.

A 30-year-old woman with a history of recurrent abdominal pain for the past 20 years presented to the emergency department with complaints of new-onset abdominal pain for one day. The pain was located in the umbilical and left lumbar region. It was rated eight out of 10 in severity and characterized as colicky. The pain was non-radiating, did not relate to food or posture, and did not respond to over-the-counter analgesics. Past episodes of pain were often unpredictable and occurred roughly every two weeks, resulting in frequent hospital admissions, numerous blood tests, endoscopic examinations, and abdominal imaging. The list of presumed diagnoses included gastroenteritis, peptic ulcer disease, pancreatitis, urinary tract infection, pelvic inflammatory disease, cholecystitis, and appendicitis. These episodes did not fully respond to any oral or injectable antibiotics, analgesics, or antispasmodics but typically resolved on their own within two to five days. During some of these episodes of pain, she had recurrent non-pitting, non-pruritic swelling in her upper and lower extremities. (Figures 1, 2) Less frequently, she also experienced lip, tongue, eyelid, and facial swelling (Figure 3). Additionally, she experienced severe dyspnea that required admission to the intensive care unit (ICU) on two occasions. There was no record of similar illnesses in her family members.

Her vital signs were stable at presentation, except for tachycardia. The physical examination was unremarkable for the chest and cardiovascular system. However, the abdomen was distended and tender in the umbilical and left lumbar regions. Laboratory tests indicated mild neutrophilic leukocytosis, with normal renal function tests, liver function tests, serum amylase and lipase.

**Figure 4:**
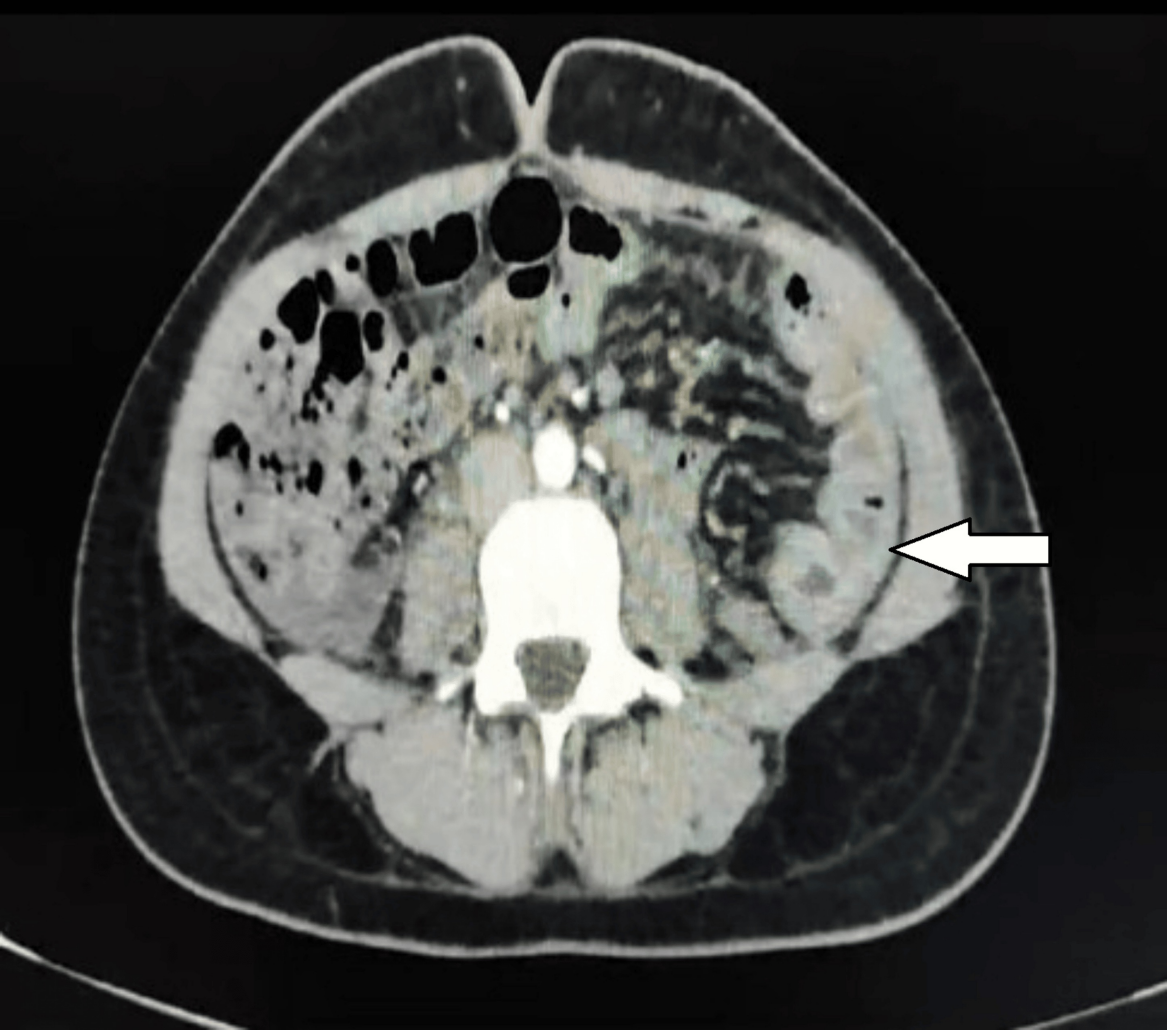
Edema of the colonic wall The white arrow depicts edema of the sigmoid colon. Photo taken four weeks prior, during the last episode.

**Figure 5:**
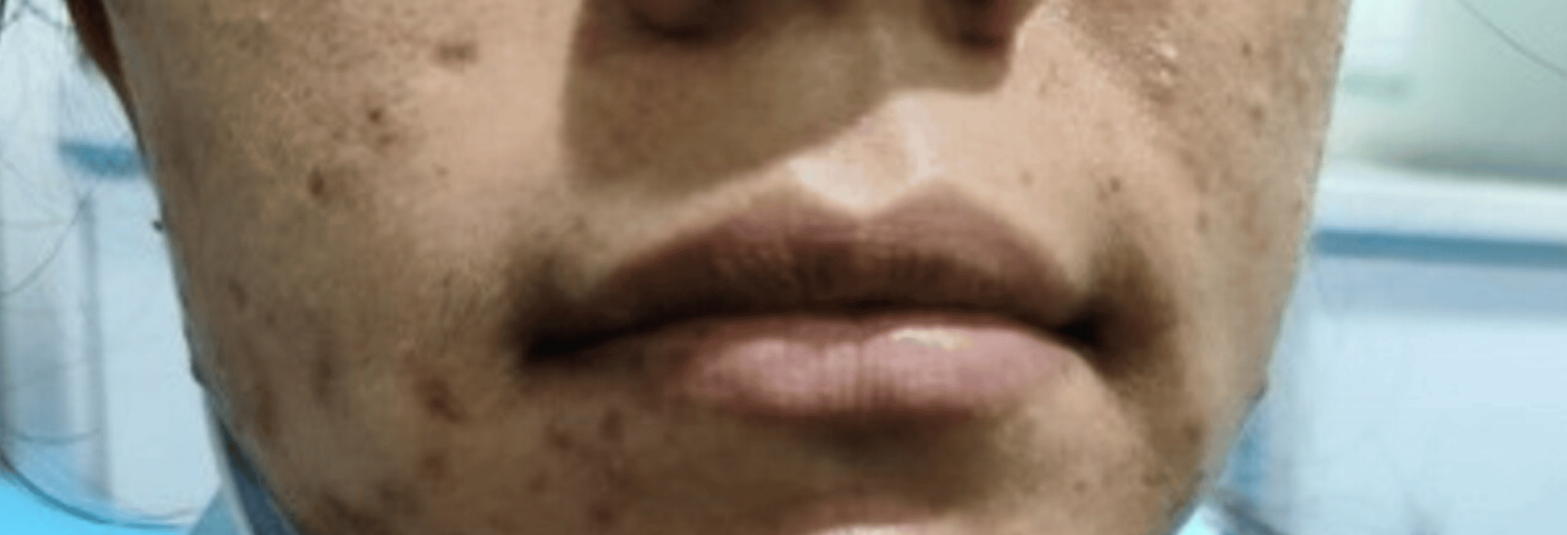
Swelling of the face and lip subsided after plasma-derived C1-INH therapy C1-INH: C1 esterase Inhibitor; photo taken seven weeks back, after the acute episode subsided.

A contrast-enhanced computed tomography (CECT) scan of the abdomen revealed significant edema in the wall of the descending and sigmoid colon (Figure 4). Subsequent laboratory tests indicated a C4 complement level of 0.5 mg/dl (normal range: 10-40 mg/dl) and a markedly reduced level of C1-INH at 0.0259 g/l (normal range: 0.21-0.39 g/l). Genetic testing confirmed a pathogenic heterozygous variant of exon 8 of the SERPING1 gene, leading to the diagnosis of type I HAE. The patient underwent treatment with intravenous plasma-derived C1-INH, administered at a dose of 500 U once a week and on an as-needed basis. After one month of therapy, she reported a significant improvement in her symptoms, including pain and angioedema, noting a reduction in both the frequency and severity of her condition (Figure 5).

## Discussion

HAE is a rare hereditary disorder with a prevalence of approximately 1:50,000 to 1:100,000 [2]. Heinrich Quincke first described the disease in 1882, and William Osler documented its heritable nature in 1888 [2,3]. In 1963, Donaldson and Evans found that HAE is a genetic disorder of C1-INH deficiency [4].

HAE has no differences in prevalence based on ethnicity or sex, but it tends to be more severe in females. Approximately three-fourths of patients have a positive family history, which indicates autosomal dominant inheritance, while 25% result from de novo mutations [5]. There are three main types of HAE: Type I, Type II, and Type III. Type I HAE, the most common form (85% of cases), is marked by a deficiency of C1-INH. Type II HAE involves a dysfunction of C1-INH. In contrast, type III HAE is typically estrogen-dependent with normal C1-INH activity. Type I and II HAE result from a mutation in the SERPING1 gene, which encodes C1-INH. The C1-INH plays a crucial role in the coagulation, complement, contact systems, and fibrinolysis. A deficiency in C1-INH results in elevated levels of plasmin, which facilitates the breakdown of bradykinin from high molecular weight kallikrein. Bradykinin binds to the B2 receptor on endothelial cells, producing cGMP, prostacyclin, and nitric oxide, which causes smooth muscle relaxation, vasodilation, increased vascular permeability, and edema [1,4,6,7].

All three types of HAE present in similar ways and can be life-threatening, especially when laryngeal involvement occurs. The most common symptom is diffuse skin swelling that is non-pitting and non-itchy, typically affecting the extremities, face, neck, lips, and oral cavity. This is often followed by episodes of recurrent, unexplained abdominal pain, which may occur with or without nausea, vomiting, and diarrhea [3,5,8]. Two episodes of laryngeal edema and multiple instances of non-pitting edema in the lips, eyelids, and face, along with recurrent abdominal pain, were observed in this patient. Some attacks may have a preceding trigger like trauma, medical procedures, stress, oral contraceptives (estrogen), infectious processes, and angiotensin-converting enzyme (ACE) inhibitors. Most HAE cases arise in childhood or early adulthood, with many patients enduring recurrent episodes before diagnosis, leading to unnecessary consultations, hospitalizations, and surgeries [1,7,9,10].

Acute treatment of HAE includes intravenous infusions of plasma-derived C1-INH (pdC1-INH), recombinant human C1-INH (rhC1-INH), icatibant (bradykinin B2-receptor antagonist), or ecallantide (recombinant plasma kallikrein inhibitor). Our patient was also treated with an intravenous infusion of plasma-derived C1-INH. Short-term prophylaxis is recommended for major dental work, surgery, intubation, endoscopies, and stressful events. Long-term prophylaxis depends on attack severity and frequency, quality of life impact, treatment access, and comorbid conditions. First-line therapies include monoclonal plasma kallikrein inhibitor (lanadelumab) and intravenous or subcutaneous pdC1-INH. Second-line options are anabolic androgens or antifibrinolytics like tranexamic acid, used for both acute and prophylactic treatment, despite some controversy [1-3,7,8].

## Conclusions

This case illustrates the challenges in diagnosing and managing HAE, particularly in patients without a family history and in resource-limited settings. It underscores the importance of increased awareness among healthcare providers to prevent unnecessary investigations and treatments, including surgeries. Given the rarity of HAE, its diverse manifestations, and the extended timeframe required for diagnosis, we report this case to emphasize the importance of early diagnosis and effective management to alleviate patient suffering.
